# Irradiation-induced polymorphism in Fe–Cr alloys

**DOI:** 10.1038/s41598-025-22150-8

**Published:** 2025-10-08

**Authors:** Ebrahim Mansouri, Xiaoqing Li, Pär Olsson

**Affiliations:** 1 Nuclear Science and Engineering, Department of Physics, KTH Royal Institute of Technology, Stockholm, SE 10691 Sweden; 2 Department of Mechanical and Materials Engineering, Queen’s University, Kingston, ON K7L 3N6 Canada; 3 Applied Materials Physics, Department of Materials Science and Engineering, KTH - Royal Institute of Technology, Stockholm, SE 10043 Sweden

**Keywords:** Irradiation-induce damage, C15 Laves phase, Non-linear magnetic behavior, Fe–Cr alloys, Metals and alloys, Atomistic models

## Abstract

Direct damage evolution simulations based on electronic structure physics show a significant correlation between Cr concentration and polymorphism in the form of localized formation of C15 Laves phase structures in Fe–Cr alloys under irradiation. We elucidate the role of Cr content in the formation and stabilization of the C15 Laves phase structure, which is crucial to understanding the behavior of materials under extreme conditions. This study also reveals a connection between non-linear magnetic behavior and irradiation-induced swelling in Fe–Cr alloys. These results advance the comprehension of radiation-induced changes in magnetization and suggest a novel experimental approach for detecting C15 clusters in irradiated Fe–Cr alloys.

## Introduction

Fe–Cr alloys play an essential role in nuclear reactor technology due to their remarkable combination of properties^1,2^. The alloying of chromium (Cr) in Fe–based alloys improves high-temperature oxidation resistance^3^ and suppresses void swelling under irradiation^4^, making these alloys critical for next-generation reactor designs. However, radiation-induced microstructural evolution, particularly the formation of nanoscale defect clusters, remains a fundamental challenge in predicting long-term material performance.

While classical radiation damage theory posits an evolution from point defects to low-dimensional clusters and ultimately extended dislocation loops^5^, recent investigations are revealing a more complex picture involving the transient stabilization of three-dimensional nanoscale phases^6,7^. Notably, the formation of the C15 Laves phase as an intermediate stage in irradiated body-centered cubic (bcc) Fe has been demonstrated through quantum mechanical simulations^8,9^. These metastable C15 clusters, distinct from conventional planar defects, exhibit unique mechanical and magnetic properties that can influence microstructural development, particularly in the early stage of irradiation doses. Intriguingly, similar phenomena appear to extend beyond bcc systems. Emerging evidence, supported by advanced computational techniques, suggests that face-centered cubic metals also exhibit a propensity to form compact 3D nano-phase inclusions, such as the A15 Frank-Kasper phase, prior to dislocation loop nucleation^7^. Understanding the atomic-scale mechanisms governing C15 Laves phase formation, and how its stabilization depends on Cr content in irradiated Fe–Cr alloys, is therefore essential for developing radiation-resistant alloys.

To investigate this phenomenon, we employed the creation-relaxation algorithm (CRA) driven by electronic structure physics to theoretically represent irradiation-induced damage. The CRA model, which was originally introduced by Limoge et al. in the late 1980s^10^, has been widely adopted by various researchers to study the evolution of irradiation-induced microstructures in different model materials^6,9,11–13^. While previous studies have applied the CRA framework within classical Molecular Dynamic (MD) to explore large-scale microstructure evolution and amorphization phenomena, these approaches remain limited by the accuracy and transferability of empirical interatomic potentials. Such potentials often fail to capture the complex interplay between electronic structure, chemical bonding, and magnetism, particularly in chemically complex, magnetic or correlated systems. In contrast, our density functional theory (DFT)-driven CRA directly resolves atomic relaxations and energetics from quantum mechanical principles, enabling explicit consideration of localized electronic and magnetic effects that govern defect formation, migration, and stability. Although computationally restricted to smaller systems, this approach offers atomistically resolved insights into radiation-induced defect processes that are inaccessible to classical MD.

The CRA model progressively introduces point defects, specifically Frenkel pairs (FPs), into a pristine crystal lattice to simulate amorphization or radiation damage in the solids. In the latter case, the target irradiation dose was achieved by correlating the total number of inserted FPs ($N_\textrm{FP}$) with the total number of lattice sites (*N*), expressed as the displacement per atom (dpa). The irradiation dose was assessed according to the canonical displacement per atom^12^, defined as the ratio of the inserted FP number to the total number of lattice sites ($\phi = {N_\textrm{FP}/N}$). Regardless of Cr content, the simulation cell used in this study contained 1024 lattice sites. The CRA was employed to simulate low-energy irradiation damage, mimicking electron irradiation at very low temperatures where thermal diffusion is negligible. In this regime, radiation-enhanced diffusion may still play a role due to the high concentration of point defects generated by irradiation (i.e. CRA), even in the absence of thermal activation. Here, FPs were introduced into each simulation cell, and the evolution of the resulting defects is driven by the induced stress and strain fields. All modeled materials were subjected to approximately 350 FP insertion events, corresponding to a cumulative irradiation dose of $\sim$ 0.35 dpa.

In this study, we employ DFT-based simulations to explore the influence of the Cr concentration on the irradiation-induced microstructural evolution in Fe–Cr alloys, with a focus on the formation and stabilization of C15 Laves phase structures. Our findings reveal that while Cr content has minimal impact on the defect number densities, it significantly affects the size and stability of C15 clusters, thereby suppressing the irradiation-induced swelling. The observed nonlinear magnetic response under irradiation suggests a magneto-volume-driven mechanism, where Cr-induced lattice deformations and reduced system magnetization (*M*) enhance C15 phase stability. Notably, the antiferromagnetic alignment of C15 cluster moments relative to the host matrix provides a distinct magnetic signature, offering a potential experimental pathway for detecting these nanoscale structures, which are otherwise challenging to resolve via conventional microscopy. These insights deepen our understanding of the radiation-induced phase transformations and magnetic behavior in Fe–Cr alloys, paving the way for novel experimental approaches in material characterization under extreme conditions.

## Results and discussion

We initially examined the relationship between the Cr content and the irradiation-induced volume expansion (swelling) in Fe–Cr alloys, alongside the alloying effect on the formation and stability of the defect clusters, particularly the non-parallel three-dimensional C15 Laves phase structure.

Swelling was estimated indirectly by calculating the ratio of global pressure change ($\Delta {P}$) to the bulk modulus (*B*), i.e., $\Delta P/B$^14^, derived from ionic relaxation (IR) calculations, where the supercell volume was fixed. Bulk moduli for each Fe–Cr system were selected based on experimental data from the literature^15,16^. Swelling was also monitored directly via relative volume change ($\Delta V/V_0$) from full relaxation (FR) calculations, where both the supercell volume and ionic positions were relaxed (Fig. S1 in Supplementary Material).

**Fig. 1:**
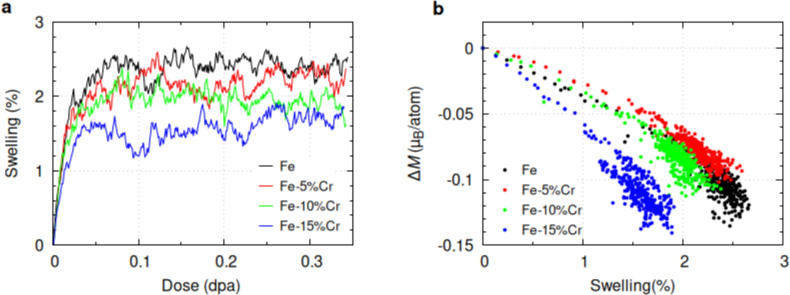
a) Dose-dependent evolution of averaged irradiation-induced swelling in Fe alloys. b) Averaged change in global *M* per atom relative to corresponding swelling in various Fe and Fe–Cr systems.

First-principles calculations consistently predict a two-stage swelling evolution across the different Fe–based compounds (Fig. 1a). In this study, swelling refers specifically to different volumetric strain induced by the accumulation of irradiation-generated point defects (i.e. vacancies and interstitials) and small defect clusters. These were introduced through the CRA method and evaluated via pressure-based and volume-based metrics. Other experimentally significant swelling contributions such dislocation loop evolution and void growth are not included in this atomistic modeling framework and remain beyond the scope of the present analysis.

As shown in (Fig. 1a), swelling initially increases linearly due to the rapid accumulation of point defects generated by the CRA (Figs. S4 and S5 in the Supplementary Material). These defects exhibit distinct relaxation volumes: self-interstitial atoms (SIAs) have positive anisotropic volume relaxation (ranging from 18.17 to 19.86 Å$^{3}$, depending on the type of SIA^17^), while vacancies possess a negative isotropic small relaxation volume, with values of 3.04 Å$^{3}$ for Fe and 6.67 Å$^{3}$ for Cr vacancies^17^. This interplay between the positive and negative relaxation volumes leads to an overall volume expansion or swelling. However, at low irradiation doses, around 0.05 dpa, steady-state saturation emerges as the stress fields of the defect clusters become constant (Fig. S2b). These fields, mediated by athermal interactions, influence the growth of the defect clusters and potentially promote spontaneous local recombination of closely situated Frenkel pairs or their coalescence into more complex configurations during relaxations^12^.

Furthermore, as shown in Fig. 1a, a notable correlation exists between Cr content and the saturated swelling in Fe-based alloys beyond 0.05 dpa. For instance, irradiation-induced swelling in pure Fe is approximately 40% higher than in Fe-15 at.% Cr. Experimental studies under high-temperature and extensive irradiation conditions have demonstrated that even small Cr additions ($\sim$ 3 wt.%) significantly reduce peak swelling in high-purity Fe–Cr alloys^4,18,19^. Pure Fe exhibited a maximum swelling of approximately 1%, while a relative reduction of approximately 90% was observed for other Fe–Cr alloys, highlighting the substantial impact of the Cr concentration in mitigating swelling induced by irradiation. This reduction in void swelling has been attributed to cavity formation, which was drastically reduced with increasing Cr content, rather than to the diffusivity of SIA clusters in the Fe–Cr alloys, as described by Terentyev et al.^20^.

In the absence of thermally activated SIA diffusion and given the uniformity in vacancy cluster size and density across Fe–Cr systems (Figs. S4, S6 in Supplementary Material), the diverse magnetic behavior of these systems may explain their differing responses to irradiation. To explore this, we investigated the relationship between total magnetization change ($\Delta {M}$) and swelling using DFT-driven CRA model calculations, including ionic and full relaxation techniques (see Fig. S1b in supplementary data). It is worth mentioning that although thermal diffusion is suppressed in our study, a contribution from radiation-enhanced diffusion due to irradiation-induced defect accumulation cannot be entirely ignored, as excess point defects generated by displacement events can promote atomic mobility even at low temperatures^21^.

As shown in Fig. 1b, Cr content strongly influences the change in global *M*, reflecting the varied responses of Fe-based alloys to irradiation. Higher Cr content reduces ferromagnetic (FM) ordering, weakening ferromagnetism and the magneto-volume effect (Fig. S3 in Supplementary Material). This reduction may arise from the differing relaxation volumes of Fe and Cr defects, as demonstrated by Wróbel et al.^17^. Their DFT calculations show that the alloying environment significantly influences the magnetic properties, formation energies, and relaxation volumes of point defects in bcc Fe, Cr and their special quasi-random structures (SQS). Specifically, in binary SQS-FeCr alloys, defects with smaller relaxation volumes exhibit larger negative $\Delta {M}$ values^17^, consistent with other studies^22–24^. Additionally, our DFT-FR calculations reveal a nonlinear relationship between Cr content and swelling (Fig. S1). While $\Delta {M}$ initially drops linearly, it eventually saturates, with data points scattering around the final swelling states (see also Fig. 1). Notably, Fe–Cr alloys exhibit distinct magnetization responses compared to pure Fe. For instance, adding 5 at.% Cr suppresses changes in total *M*, while higher Cr concentrations accelerate the reduction in *M* and swelling rates beyond those of pure Fe.

The Supplementary Material outlines the dose-dependent evolution of the vacancies, interstitials, and their clusters. Notably, the defect densities and cluster sizes (both average and largest) show no correlation with Cr content. However, the number density of isolated surviving vacancies is twice that of mono interstitials, indicating a greater tendency for interstitials to form larger clusters. This aggregation occurs even without thermally activated diffusion, highlighting the significant role of irradiation-induced stress fields in defect evolution, as reported elsewhere^12,13^. While vacancy and interstitial populations and their cluster sizes remain independent of Cr content, the formation energy, local magnetic moments, size, and stability of three-dimensional clusters, particularly C15 Laves phases, exhibit a strong dependence on Cr concentration. This underscores the importance of investigating the formation and stability of constrained defect clusters, such as C15 structures in Fe–Cr systems.

**Fig. 2:**
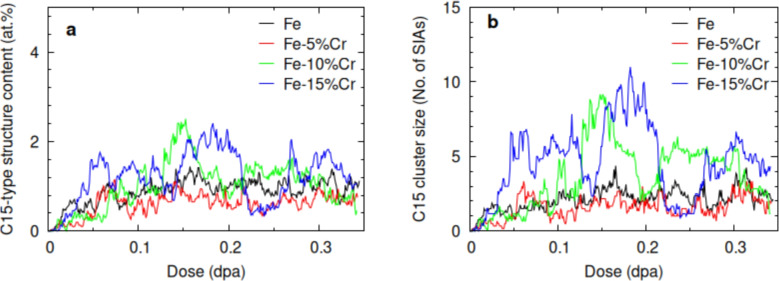
a) Population of C15-type atomic structure and b) the largest C15 Laves phase structures formed in the Fe and Fe–Cr alloys as a function of irradiation dose. Color references can be found in the online version of the article.

First-principles^9,25^ and classical molecular dynamics studies^13,26,27^ predict C15 Laves phase formation in irradiated bcc Fe. Here, we show that both imperfect and perfect C15-type structures, including clusters of triangular and/or hexagonal di-interstitial rings, are likely to form in irradiated Fe-based alloys. This structural diversity highlights the irradiation-induced polymorphism of the C15 phase under these conditions in Fe–Cr systems, aligned with previous finding^8,9^. Moreover, increasing Cr concentration stabilizes these features, facilitating the development of large perfect C15-type structures, as most clearly observed in the Fe-15% Cr alloy. These findings are supported by our first-principles predictions under the applied irradiation conditions in this study (see Fig. S7 in Supplementary Material). Figure 2 shows the dose-dependent evolutions of the atomic C15-type structures and the emergence of the largest C15-type clusters in the damaged bcc Fe and Fe–Cr alloys with varying Cr contents. An increase in the C15-type structure content (Fig. 2a) is observed with increasing antiferromagnetic (AF) alloying element of Cr, which potentially facilitates the formation of giant C15-type clusters (Fig. 2b).

**Fig. 3:**
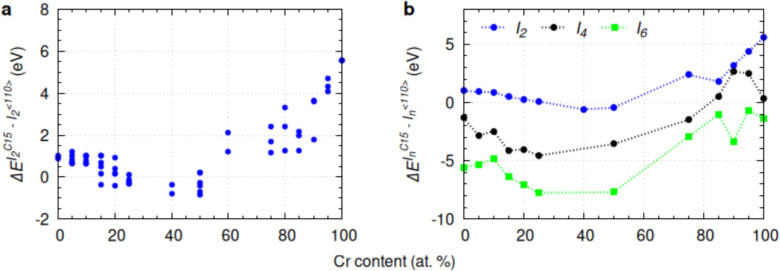
Formation energy difference between a) two-SIA clusters ($I_2$) and b) $\textit{n}$-SIA clusters ($I_n$) in C15 and $\langle 110\rangle$-dumbbell configurations as a function of Cr content.

Consistent with previous studies on C15 Laves phase stability^25,26^, we performed separate DFT calculations, independent from the DFT-driven CRA simulations, to compare the formation energy, magneto-volume behavior, and local magnetic moments of these structures in pure Fe and various SQS $\hbox {Fe}_{1-x}\hbox {Cr}_{x}$ alloys. In these calculations, C15 clusters were randomly incorporated into each SQS-FeCr alloy (Fig. S8 in Supplementary Material). The formation energy of a C15 cluster (equivalent to 12 SIAs and 10 vacancies, or a net inclusion of two SIAs, $I_{2}$) is higher than that of the most stable $\langle 110\rangle$-dumbbell configuration in pure Fe. However, as Cr content increases, the energy difference between C15 ($I^{C15}_\textrm{2}$) and $\langle 110\rangle$-dumbbell ($I^{\langle 110\rangle }_\textrm{2}$) configurations, $\Delta {E}^{{I^{C15}_\textrm{2}}-{I^{\langle 110\rangle }_\textrm{2}}}$, decreases from $\sim$ 1 eV for pure Fe to $\sim$ 0.4 eV for Fe-15 at.% Cr, a 60% reduction. This highlights the increased tendency for SIAs to form C15 clusters in Fe–Cr alloys, as shown in Fig. 3.

The scattered data points in Fig. 3a reflect the randomized inclusion of C15 clusters at various locations within each SQS-FeCr alloy. Notably, the formation energy of C15 clusters becomes lower than that of $\langle 110\rangle$-dumbbells in Fe–Cr alloys with 15–50 at.% Cr, depending on local Cr distributions. In contrast, C15 clusters in pure Cr exhibit a high formation energy of 5.5 eV, suggesting optimal Cr content for C15 stabilization lies between 15–50 at.%. While bulk Fe–Cr alloys with 15–50 at.% Cr are within the miscibility gap and are unstable under equilibrium conditions at low enough temperature, such local concentrations may develop during irradiation due to radiation-induced segregation or local enrichment at sinks such as grain boundaries, dislocations, or void surfaces. Our DFT calculations provide insights into the relative stability of defect configurations within these potential non-equilibrium, Cr-rich environments.

**Fig. 4:**
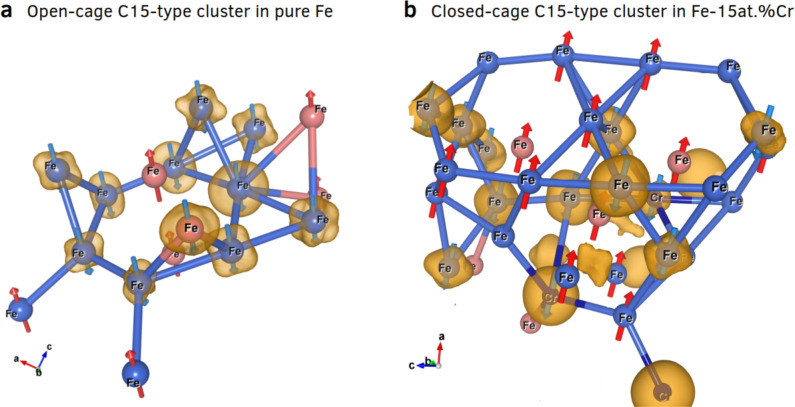
Comparison of a) an open-cage and b) a three-fold closed-cage C15 clusters formed in damaged bcc Fe and Fe-15 at.% Cr, respectively. The orange clouds, enveloping atoms with spin-down orientation (blue arrows), represent the three-dimensional spin density isosurfaces of atoms that have either undergone spin flip or exhibit AF ordering. Red arrows symbolize the spin-up orientations. Pink spheres represent host lattice atoms, while blue spheres without attached arrows denote atoms that have undergone spin quenching. For color references, the reader is referred to the web version of the article.

Analysis of CRA trajectories reveals that large C15 clusters form preferentially in Fe–Cr alloys with higher Cr content. In pure Fe, C15 clusters are small open-cage structures with triangular or hexagonal di-interstitial rings (Fig. 4a). In contrast, larger closed-cage C15 clusters form in Fe-15% Cr (Fig. 4b) and Fe-10% Cr (Fig. S7c in Supplementary Material). These DFT-driven CRA simulations further highlight that the irradiation-induced polymorphism of C15-type structures becomes more pronounced with increasing Cr concentration in the Fe–Cr system.

To further explore the relationship between C15 formation energy and Cr content, we incorporated *n*-SIA C15 clusters ($I^{C15}_\textrm{n}$) into SQS-FeCr systems and compared their formation energies with equivalent $\langle 110\rangle$-dumbbell clusters ($I^{\langle 110\rangle }_\textrm{n}$). As shown in Fig. 3b, larger C15 clusters are energetically more favorable than parallel dumbbells, particularly in Fe–Cr alloys with 15–50 at.% Cr. This stabilizing effect is consistent with previous findings for pure Fe^25,26^. Detailed formation energy and magnetization analyses are provided in Section VI of the Supplementary Material.

**Fig. 5:**
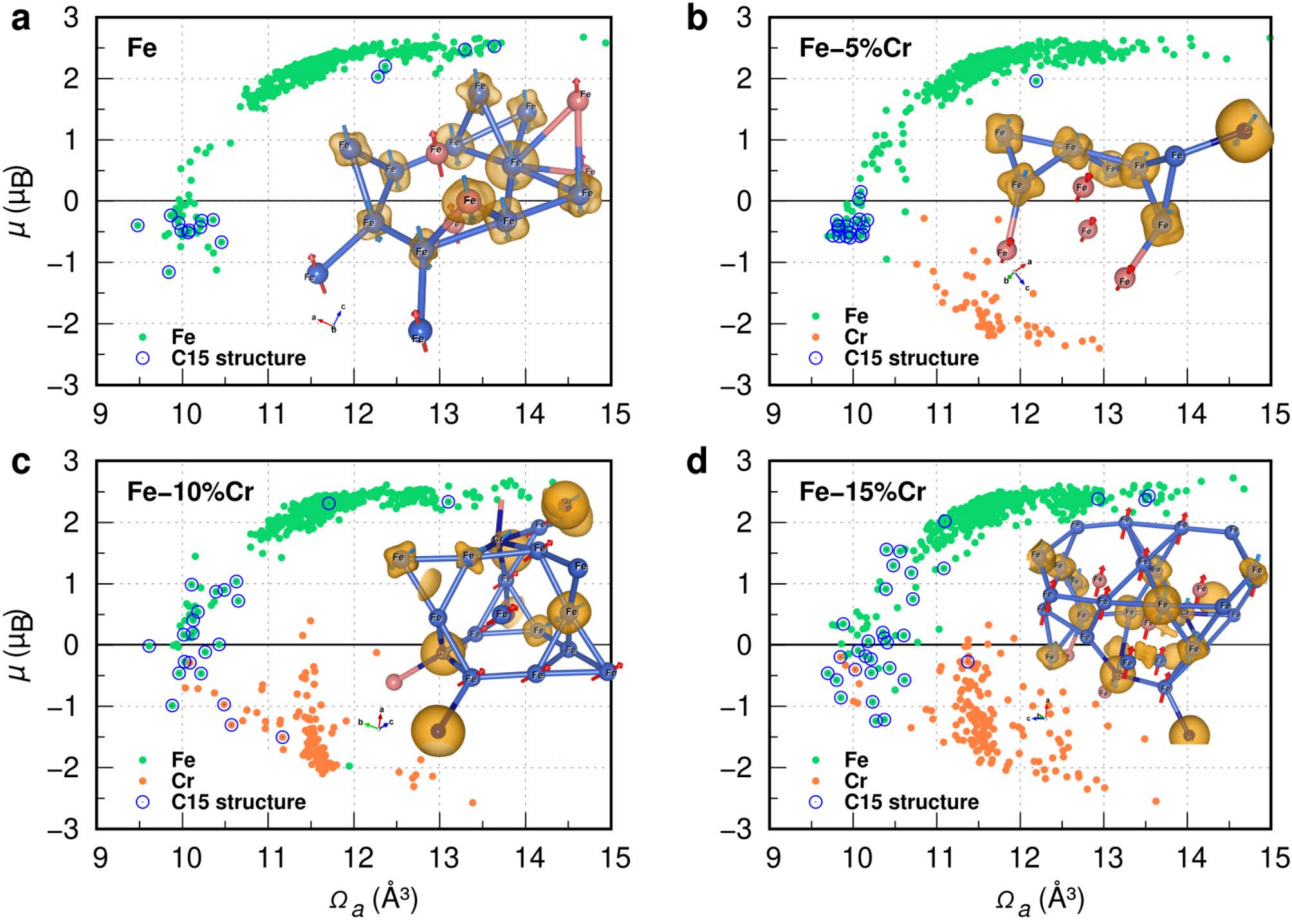
Distribution of local $\mu$ of atoms in relation to their local $\Omega _{a}$ in a) pure Fe, b) Fe-5 at.% Cr, c) Fe-10 at.% Cr, and d) Fe-15 at.% Cr. Blue circles highlight data points representing the C15 Laves phase structure formed in damaged bcc Fe and Fe–Cr systems. Green and orange data points indicate the $\mu$-$\Omega _{a}$ relationships for Fe and Cr, respectively. The C15 Laves phase structures present in each Fe–Cr system are included in the corresponding $\mu$-$\Omega _{a}$ plots for comparison.

Further analysis of C15 clusters in irradiated Fe–Cr systems reveals that a local Cr concentration of 15–40 at.% within or adjacent to the cluster is critical for stabilization. For example, the highest energy gain ($\sim$ 15 eV) occurs when SIAs form C15 clusters with 15 at.% Cr. These clusters mitigate radiation-induced stress by reducing hydrostatic pressure as Frenkel pair density saturates. This could be attributed to strain field relaxation and the elastic properties of the C15 phase, rather than intrinsic chemical preferences, as supported by coherent potential approximation (CPA) calculations (Fig. S9 in Supplementary Material). Additionally, DFT-CRA simulations show a clear attraction between Cr atoms and SIA defects within C15 clusters. For example, in Fe-10%Cr, the local Cr content of C15 clusters is approximately 20% (Fig. S7c). In Fe-15%Cr, large C15 clusters contain around 10–12% Cr, whereas smaller, transient clusters can initially reach local Cr concentrations of up to $\sim$ 33%, illustrating an extreme case of Cr enrichment around SIAs. In contrast, in Fe-5%Cr, the Cr content in C15 clusters remains mostly below 5%, with only a single transient cluster exhibiting $\sim$ 6.6% Cr. Cr-vacancy configurations are also observed but are less persistent and more spatially dispersed. The Cr content of C15 clusters in their largest state is highlighted by the circled orange dots in Fig. 5.

Furthermore, the CPA calculations (Fig. S9 in Supplementary Material) show no thermodynamic driving force for Fe–Cr segregation in C15 structures. DFT simulations predict limited stability for large C15 clusters in Fe, which transform into small dislocation loops with $\langle 110\rangle$ Burgers vectors^28^. The bulk modulus of C15 Fe (152 GPa) is lower than that of bcc Fe (173 GPa), indicating that the C15 phase is a soft structure that forms under irradiation-induced pressure but becomes unstable as it grows.

Due to their small size and transient nature, C15 clusters are challenging to detect experimentally using techniques like transmission electron microscopy (TEM)^8,25,28^. Their formation and behavior are driven by local stress fields and lattice distortions, rather than inherent chemical preferences under irradiation.

As noted in other studies^8,9^ on pure Fe, the C15-type structures antiferromagnetically aligned with the bcc host Fe atoms while exhibiting short-range ferromagnetism, as initially reported in^25^. However, herein, as demonstrated in Figs. 5a–d, the alloying of Cr atoms weakens the short-range ferromagnetism of C15 cluster in host bcc Fe and transforms its magnetic state into locally short-range antiferromagnetism in Fe–Cr alloys for higher atomic concentration of Cr. To elaborate this, the local magnetic moments of all 1024 atoms are plotted versus their related local atomic volumes (Voronoi cell) for each Fe and Fe–Cr alloys in Fig. 5, at irradiation doses between 0.15 - 0.2 dpa, where the C15 Laves phases appear.

As shown elsewhere^8,25,26,29^, the atoms that form C15 clusters tend to pair antiferromagnetically with the FM atoms of the host Fe lattice under constrained configurations. However, introducing Cr atoms weakens the local FM ordering of the C15-type structure and establishes a local AF ordering of atoms forming C15 clusters. Although the CPA calculation (see Fig. S9 in the Supplementary Material) does not predict the formation of the C15 Laves phase under equilibrium conditions, regardless of Cr concentration, non-equilibrium processes such as high-pressure conditions induced by irradiation may favor its formation^8^. Additionally, the accumulation of SIAs in a C15 configuration could potentially stabilize the C15 Laves phase by mitigating lattice distortions. As a result of these effects, C15 clusters exhibit spin flip or quenching, leading to a locally averaged magnetic moment of approximately -0.7 $\mu _\textrm{B}$. Given their unique magnetic properties, C15 clusters could be experimentally identified through advanced magnetic imaging techniques in conjunction with TEM^30^.

The difference in behavior of C15 clusters in Fe and Fe–Cr alloys could be related to the different magneto-volume effects of the systems, resulting from the different relaxation volumes of Fe and Cr vacancies and SIAs. As described by Wróbel et al.^17^ using DFT calculations, the alloying environment significantly affects the magnetic properties and atomic-level distortions (elastic dipoles and relaxation volumes) of point defects in bcc Fe, bcc Cr, and their disordered alloys. This influence arises from variations in the local volume and local moment of defects containing Fe–Fe and Fe–Cr dumbbells, as also reported in other studies^22–24^. Furthermore, variations in the magnitudes of magnetic moments associated with defects significantly affect the formation energy of these defects. With DFT ionic and full relaxation calculations, it has been demonstrated that the relaxation volumes and formation energies of SIA in dumbbell configurations within disordered Fe–Cr alloys decrease with increasing Cr content. Furthermore, as shown by Marinica et al.^25^ and Dézerald et al.^26^, C15 clusters containing four or more SIAs have lower formation energies than the equivalent parallel configuration. In addition, the formation energy of dumbbells decreases with increasing Cr content in SQS-FeCr alloys. Together, these results support the hypothesis of stabilization of the C15 cluster with increasing Cr content proposed in this study.

## Methods

In this study, several aspects of irradiation-induced degradation were investigated within the DFT framework. Energy minimization calculations were conducted using the creation-relaxation algorithm to simulate direct damage insertion in a lattice^9^. We employed the projector-augmented plane wave method^31^ implemented in the Vienna *ab initio* simulation package (VASP)^32–35^. The conjugate gradient method^36^ was used for total energy minimization after each Frenkel pair insertion. The exchange correlation term was treated using the generalized gradient approximation, as parametrized by Perdew, Burke, and Ernzerhof^37,38^.

All calculations were spin-polarized, and periodic boundary conditions, along with the supercell approach, were employed. Supercells of 8 $\times$ 8 $\times$ 8 replicas of the bcc orthogonal unit cell, each containing 1024 atomic sites, were used for both Fe and Fe–Cr systems. The 1024-atom supercell size was chosen to minimize the interactions between defects under periodic boundary conditions and to ensure that the local damage structures could evolve realistically. This setup has also been validated in earlier DFT-CRA studies^9^. The Brillouin zones were sampled using the gamma point in *k*-space. To enhance model accuracy and reduce statistical fluctuations, four and three distinct CRA trajectories were used for Fe and Fe–Cr systems, respectively. Within each Fe–Cr trajectory, chromium contents of 5, 10, and 15 at.% were randomly distributed.

Two relaxation strategies were employed to balance computational efficiency and accuracy. Full relaxation provided the most accurate representation by optimizing all degrees of freedom but was computationally expensive. Therefore, ionic relaxation in the microcanonical (NVE) ensemble was primarily used for computational feasibility, with FR calculations strategically interspersed within each CRA trajectory. For volume-conserving simulations, a 250 eV cut-off energy and a self-consistent ionic convergence criterion of 1 meV were used. Fully relaxed calculations utilized a higher cut-off energy of 350 eV and a stricter ionic convergence criterion of 0.1 meV. The choice of cut-off energies in our DFT-CRA simulations was based on achieving a practical balance between computational cost and accuracy, informed by prior benchmarks and widely adopted values in radiation damage studies using VASP for Fe-based systems^23,39–41^.

To investigate the relationship between Cr content and the stability of irradiation-induced defects, particularly the C15 Laves phase structure, we compared the formation energy, magneto-volume behavior, and local magnetic moments of these clusters in pure Fe and various SQS of $\hbox {Fe}_{1-x}\hbox {Cr}_{x}$ alloys. A randomized distribution of C15 clusters within each SQS-FeCr alloy was employed to mitigate potential biases arising from the Cr distribution. This analysis involved systematic insertion of perfect C15 clusters into supercells containing 250 atoms plus *n* additional SIAs, denoted as ${I^{C15}_\textrm{n}}$, following established methodologies^25,26^. FR calculations with a 3 $\times$ 3 $\times$ 3 *k*-point mesh, a 350 eV cut-off energy, and an ionic convergence criterion of 0.1 meV were used.

The formation enthalpy of C15 Fe–Cr alloys was calculated using the exact muffin-tin orbitals method^42,43^ in conjunction with the coherent potential approximation to address chemical disorder. The CPA replaces the spatially varying crystal potential in a random material with a self-consistently determined effective medium. The primitive cell of the C15 structure (six atomic sites) was adopted, with compositions set as $\hbox {Fe}_{1-x}\hbox {Cr}_{x}$ (0 $\le x \le$ 0.20). Brillouin zone integrations were performed on a 25 $\times$ 25 $\times$ 25 *k*-point mesh, and all calculations were carried out in the ferrimagnetic state. The basis set included *s*, *p*, *d*, and *f* states.

The formation enthalpy of $\hbox {Fe}_{1-x}\hbox {Cr}_{x}$ alloy in the C15 Laves phase structure is defined as

1 $$\begin{aligned} \Delta {H(x)} = E(\textrm{Fe}_{1-x}\textrm{Cr}_{x}) - (1-x)E_\textrm{bcc}(\textrm{Fe}) - xE_\textrm{bcc}(\textrm{Cr}), \end{aligned}$$

where $E_\textrm{bcc}(\textrm{Fe})$ and $E_\textrm{bcc}(\textrm{Cr})$ are the total energies of bcc ferromagnetic Fe and bcc antiferromagnetic Cr, respectively.

Microstructure evolution was characterized using the Wigner-Seitz defect analysis capabilities of OVITO^44^. Polyhedral template matching analysis^45^ with a root-mean-square deviation value of 0.25 was used to identify different structures, including C15 clusters formed in damaged cells.

## Supplementary Information

Supplementary Information.

## Data Availability

The data presented in this paper are available upon request from the corresponding authors.
