# Supplementary material for: Irradiation-induced polymorphism in Fe–Cr alloys
Source: Sci Rep. 2025 Oct 8;15:35050. doi: 10.1038/s41598-025-22150-8 (PMC12508060; doi:10.1038/s41598-025-22150-8)
Supplement: Supplementary file 1 — Supplementary Information. [file 41598_2025_22150_MOESM1_ESM.pdf]

Supplementary Material for
Irradiation-induced polymorphism in Fe-Cr alloys

Ebrahim Mansouri,[1,2,*] Xiaoqing Li,[3] and Pär Olsson[1,†]

[1] *Nuclear Science and Engineering, Department of Physics, KTH Royal Institute of Technology, Stockholm, SE-10691, Sweden.*

[2] *Department of Mechanical and Materials Engineering, Queen's University, Kingston, ON K7L 3N6, Canada.*

[3] *Applied Materials Physics, Department of Materials Science and Engineering, KTH - Royal Institute of Technology, Stockholm, SE-10043, Sweden.*

(Dated: October 5, 2025)

* Contact author: ebrahim.mansouri@queensu.ca

† Contact author: polsson@kth.se

I Radiation-induced swelling with full relaxation calculations in Fe and Fe-Cr alloys

Figure S1 shows the irradiation-induced swelling ($\Delta V/V_0$) extracted from full relaxation (FR) calculations.

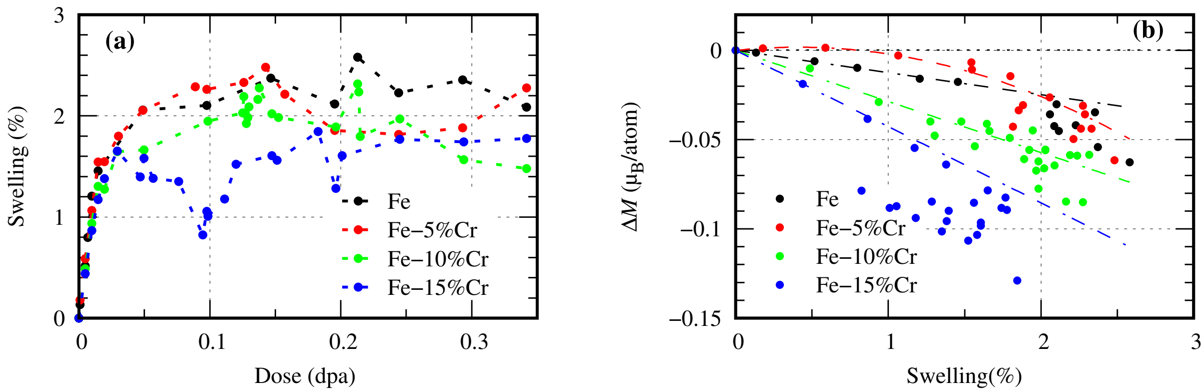

FIG. S1. a) Dose-dependent evolution of irradiation-induced swelling in Fe and Fe-Cr alloys. b) Averaged change in global magnetization (M) per atom, relative to the corresponding swelling, for various Fe and Fe-Cr alloys. In the right panel, dashed lines fitted to the data points depict instances where ΔM has not yet reached saturation values.

II Dose-dependent energy and pressure changes

Figure S2 compares the average change in energy per atom (ΔE/atom) and pressure (ΔP) as a function of displacement per atom (dpa) in bcc Fe and Fe-Cr systems within cubic supercells with side lengths of $8a_0$, where a_0 is the Fe lattice constant.

III Magneto-volume relationships in Fe alloys

Figure S3 shows how magneto-volume relationships differ among Fe-Cr systems. The bulk modulus was calculated by fitting several $E(V)$ data points to an empirical equation of state. Specifically, we employed a third-order Birch-Murnaghan equation [1]:

$$E(V) = E_0 + \frac{9V_0B_0}{16}\left\{\left[\left(\frac{V_0}{V}\right)^{2/3} - 1\right]^3 B_0' + \left[\left(\frac{V_0}{V}\right)^{2/3} - 1\right]^2 \left[6 - 4\left(\frac{V_0}{V}\right)^{2/3}\right]\right\}, \quad \text{(S1)}$$

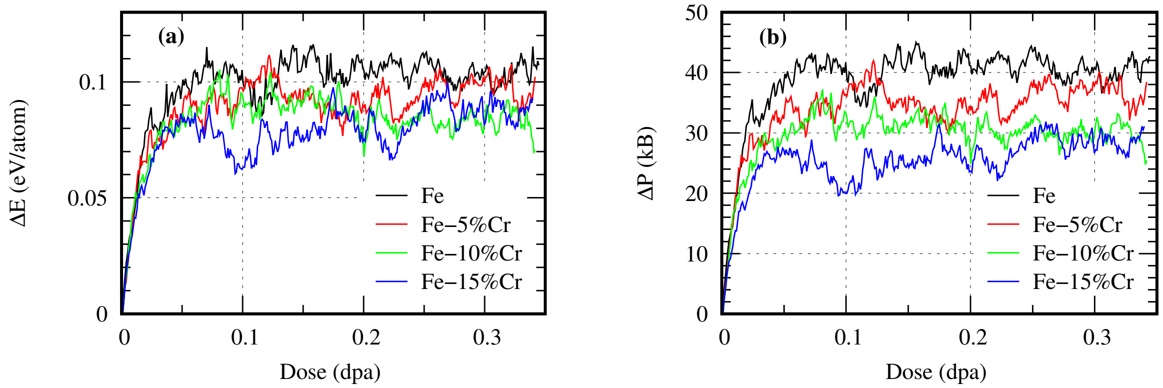

FIG. S2. Comparison of a) the dose-dependent evolution of ΔE/atom and b) ΔP in Fe alloys as a function of Cr content, extracted from ionic relaxation (IR) calculations. Color references are provided in the online version of the article.

where $E(V)$ is the energy as a function of volume, E_0 is the equilibrium energy, V_0 is the equilibrium volume, B_0 is the bulk modulus, and B_0' is the derivative of the bulk modulus with respect to pressure.

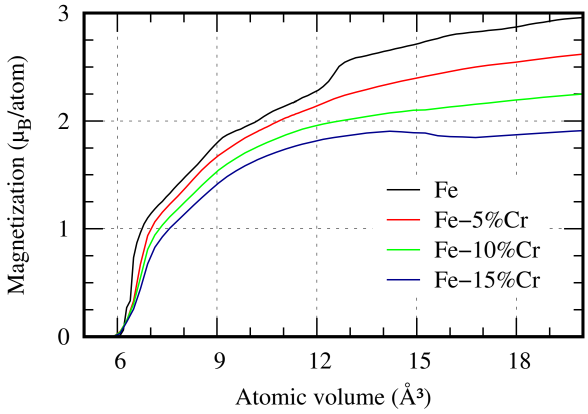

FIG. S3. Magneto-volume relationships in different Fe alloys.

IV Comparison of dose-dependent evolution of defect contents

Figure S4 shows the dose-dependent evolution of surviving Frenkel pairs (FPs), isolated interstitials (I_{mono}), and interstitial clusters (I_{C}) as a function of irradiation dose. These results are obtained from DFT-driven CRA simulations for Fe and Fe-Cr systems within supercells with side lengths of $8a_0$. Similarly, Figure S5 illustrates the dose-dependent

evolution of surviving isolated vacancies (V_{mono}) and vacancy clusters (V_{C}) as a function of irradiation dose, predicted by the same DFT-driven CRA simulations for Fe and Fe-Cr systems within cubic supercells of the same size.

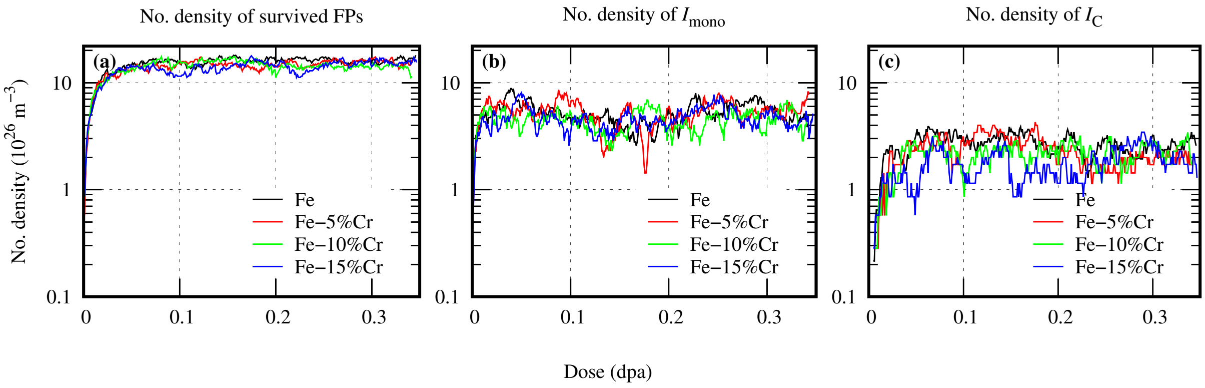

FIG. S4. Dose-dependent number density of the a) FP, b) I_{mono}, and c) I_{C} for different Fe and Fe-Cr systems.

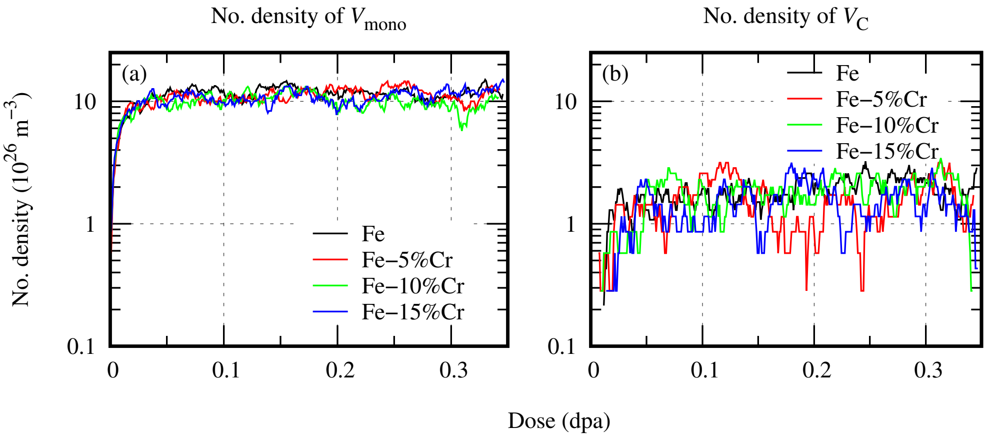

FIG. S5. Dose-dependent number density of the a) V_{mono} and b) V_{C} for different Fe and Fe-Cr systems.

Figure S6 displays the dose-dependent evolutions of the average (left panels) and largest (right panels) I_{C} and V_{C} in different Fe and Fe-Cr systems.

Table S1 summarizes the average number densities of surviving point defects in pure Fe and Fe-Cr systems. These values are obtained by averaging over all CRA trajectories and entire irradiation doses up to 0.35 dpa. In addition, table also reports the average sizes of interstitial clusters and vacancy clusters, providing insight into defect aggregation behavior.

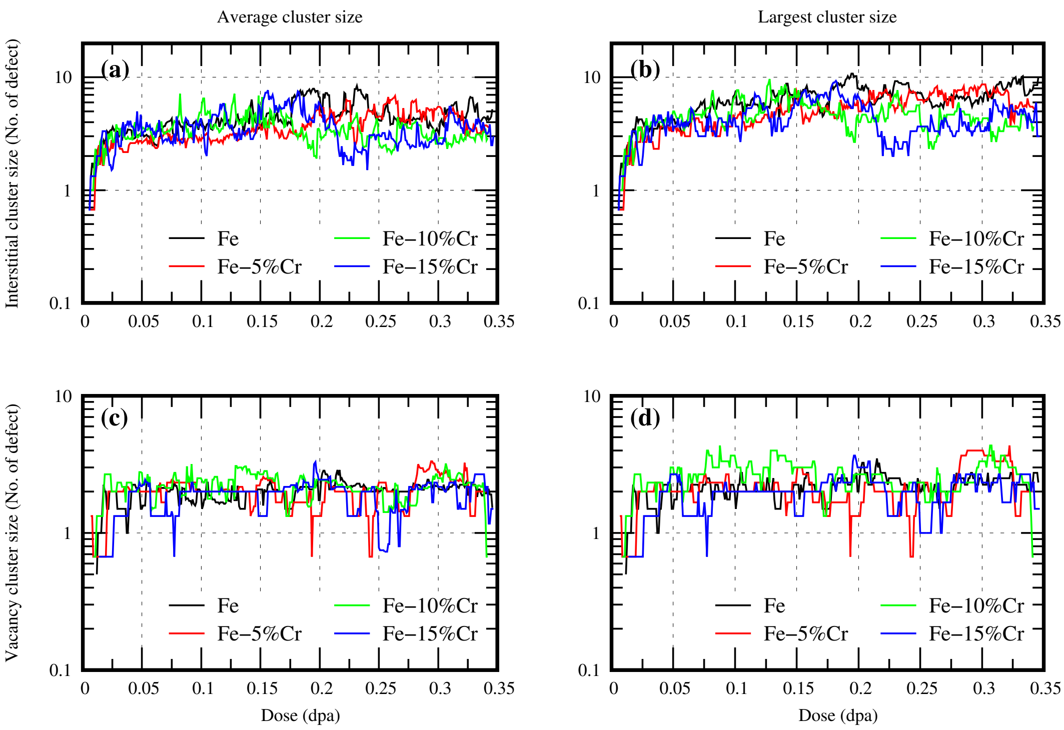

FIG. S6. Dose-dependent evolutions of the a) average and b) largest interstitial clusters along with the c) average and d) largest vacancy clusters formed in Fe and Fe-Cr alloys, resulting from DFT-CRA calculations.

All number densities are expressed in units of 10^{26} m^{-3}. This data complements the trends presented in Figs. S4 - S6 and serves to quantitatively illustrate the systematic differences in defect survival and clustering as a function of Cr concentration. These results are important for informing higher-scale models, such as rate theory or cluster dynamics, particularly in defining initial conditions for defect populations.

V The size of C15 Laves phase structure with respect to Cr contents

Figure S7 indicates the size dependency of the C15 Laves phase structure with respect to the at.% Cr in Fe and Fe-Cr alloys. As the Cr content increases, the formation and stability of such three-dimensional clusters are improved by increasing the antiferromagnetic ordering.

TABLE S1. Average number density of surviving FPs, I_{mono} and V_{mono} in pure Fe and Fe–Cr systems. Defect number densities are reported in units of 10^{26} m^{-3}. The average cluster sizes of I_{C} and V_{C}, defined as the average number of defects forming a single cluster, are also presented for comparison. All values represent averages over all CRA trajectories for each system, taken over the entire irradiation range up to 0.35 dpa.

	FP	I_{mono}	V_{mono}	Ave. size of I_{C}	Ave. size of V_{C}
Fe	15.2	5.2	11.3	4	2
Fe-5%Cr	14.2	5.3	10.9	4	2
Fe-10%Cr	13.9	4.8	9.8	4	2
Fe-15%Cr	13.6	4.8	10.7	5	2

VI Formation energies of interstitial clusters

The formation energies of an n-SIA cluster, whether in the C15 Laves phase or in the $\langle 110\rangle$-dumbbell configuration, is calculated using:

$$E_{\mathrm{f}}^{(n-\mathrm{SIA})} = E^{(n-\mathrm{SIA})} - (E_{\mathrm{ref}} + k.\mu_{\mathrm{A}} + m.\mu_{\mathrm{B}}), \tag{S2}$$

where $E^{(n-\mathrm{SIA})}$ is the total energy of a structure containing n SIAs (with k number of species A and m number of species B). E_{ref} is the total energy of the defect-free reference structure. μ_{A} and μ_{B} are the chemical potentials of atoms A and B (here, bcc Cr or bcc Fe), respectively. These chemical potentials account for the energy cost or gain associated with adding or removing atoms from the system.

The chemical potentials of each species in each random SQS-FeCr alloy with a Cr concentration below 35% were extracted directly from [2]. For higher Cr concentrations (> 35 at.%), we employed an extrapolated formula based on the data in Ref. [2]. To calculate the formation energy of SIA clusters, we followed the methodology outlined in Refs. [3, 4]. Consequently, we inserted n additional Cr or Fe atoms into a cubic 250-atom supercell to form n-SIA clusters in either C15 Laves phase or $\langle 110\rangle$-dumbbell configurations. The positions of the C15 clusters were randomly selected, as shown in Fig. S8.

Figure S9 demonstrates a linear increase in the formation enthalpy of the Fe-Cr system within the artificially imposed C15 Laves phase structure as a function of Cr concentration.

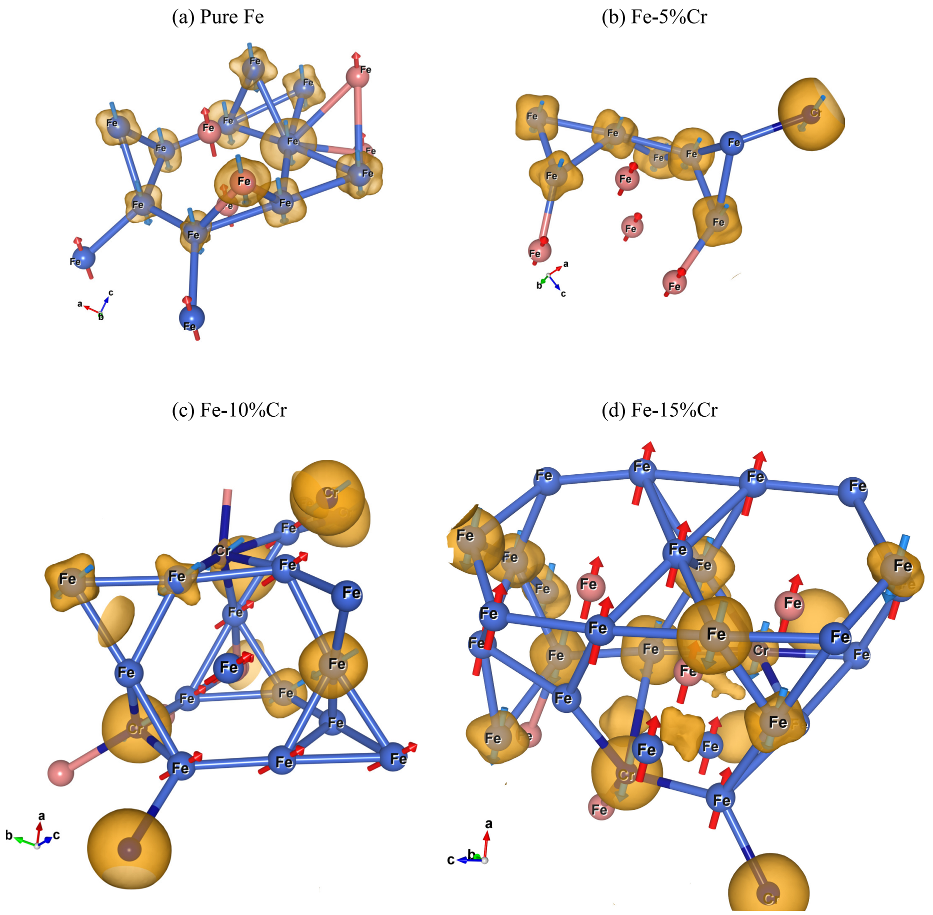

FIG. S7. Evolution of C15 Laves phase structure with increasing Cr concentration in a) Fe, b) Fe-5% Cr, c) Fe-10% Cr, and d) Fe-15% Cr as obtained from dynamic DFT-CRA calculations in cubic supercells with side length of $8a_0$. Orange clouds, enveloping atoms with spin-down orientation (blue arrows), represent the three-dimensional spin density isosurfaces of atoms that have either undergone spin flip or have antiferromagnetic ordering (Cr atoms). Red arrows symbolize spin-up orientations. Blue atoms without arrows have undergone spin quenching. Color references can be found in the online version of the article.

This trend underscores the thermodynamic instability of the Fe-Cr system in the C15 phase in the compositional range studied, indicating that this configuration becomes increasingly energetically unfavorable with higher Cr concentrations. This behavior suggests a lack of energetic driving force for the formation of the C15 Laves phase in the Fe-Cr system, aligning with the known thermodynamic preferences of Fe-Cr alloys for stable bcc phases. This thus highlights the importance of the local structures appear under irradiation conditions being

TABLE S2. The difference in formation energies of interstitial clusters in C15 Laves phase and those of ⟨110⟩-dumbbell configuration in Fe and SQS-FeCr alloys. The formation energies are in eV, and total changes in magnetization are in μ_{B}.

	$\Delta E^{I_2^{C15}-I_2^{\langle 110\rangle}}$	$\mu^{I_2^{C15}}$-μ^{bulk}	$\Delta E^{I_4^{C15}-I_4^{\langle 110\rangle}}$	$\mu^{I_4^{C15}}$-μ^{bulk}	$\Delta E^{I_6^{C15}-I_6^{\langle 110\rangle}}$	$\mu^{I_6^{C15}}$-μ^{bulk}
Fe	1	-27.9	-1.31	-40.42	-5.5	-66.77
Fe-5%Cr	0.9	-33.3	-2.38	-46.1	-5.34	-68.08
Fe-10%Cr	0.89	-31.88	-2.47	-58.21	-4.85	-68.0
Fe-15%Cr	0.47	-31.58	-4.12	-42.7	-6.41	-57.86
Fe-20%Cr	0.26	-29.77	-4.03	-38.87	-7.04	-60
Fe-25%Cr	0.09	-28.5	-4.56	-37.78	-7.76	-56.8
Fe-40%Cr	-0.59	-25.5	-	-	-	-
Fe-50%Cr	-0.42	-22.5	-3.83	-31.67	-8.0	-40.5
Fe-60%Cr	1.67	9.83	-	-	-	-
Fe-75%Cr	2.38	5.9	-1.49	21.1	-2.95	39.1
Fe-80%Cr	2.33	22.21	2.45	31.5	1.23	2.05
Fe-85%Cr	1.8	38.82	0.55	43	-1.04	-43.8
Fe-90%Cr	3.14	21.91	2.64	25.5	-3.34	17
Fe-95%Cr	4.36	5	2.51	5.3	-0.72	24.6
Cr	5.5	-1.5	0.37	0	-1.5	-3.14

stabilized by the intricate interplay of pressure, strain, and magnetic ordering.

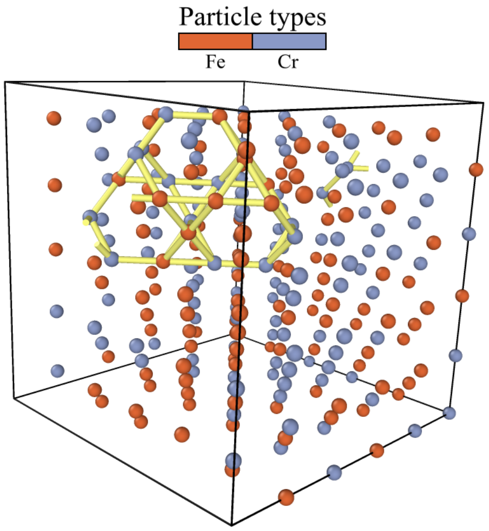

FIG. S8. A typical I_6^{C15} cluster inserted in a random position within SQS-Fe-50%Cr. The yellow bars show the virtual bounds between atoms forming the C15 structure.

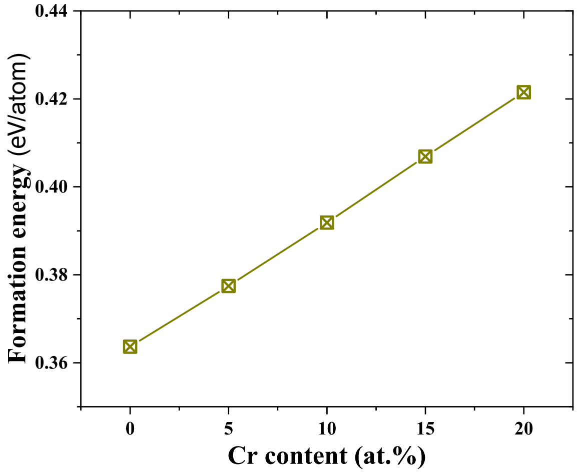

FIG. S9. Formation energy of the Fe-Cr system with C15 Laves phase structure as a function of Cr concentration, calculated using coherent potential approximation (CPA).

References

[1] Francis Birch. Finite elastic strain of cubic crystals. *Phys. Rev.*, 71:809–824, Jun 1947. doi:10.1103/PhysRev.71.809.

[2] Jan S. Wróbel, Marcin R. Zemła, Duc Nguyen-Manh, Pär Olsson, Luca Messina, Christophe Domain, Tomasz Wejrzanowski, and Sergei L. Dudarev. Elastic dipole tensors and relaxation volumes of point defects in concentrated random magnetic Fe-Cr alloys. *Computational Materials Science*, 194:110435, June 2021. ISSN 0927-0256. doi:10.1016/j.commatsci.2021.110435.

[3] M.-C. Marinica, F. Willaime, and J.-P. Crocombette. Irradiation-Induced Formation of Nanocrystallites with C15 Laves Phase Structure in bcc Iron. *Physical Review Letters*, 108 (2):025501, January 2012. doi:10.1103/PhysRevLett.108.025501.

[4] L. Dézerald, M.-C. Marinica, Lisa Ventelon, D. Rodney, and F. Willaime. Stability of self-interstitial clusters with C15 laves phase structure in iron. *Journal of Nuclear Materials*, 449 (1):219–224, 2014. ISSN 0022-3115. doi:https://doi.org/10.1016/j.jnucmat.2014.02.012.
